# Editorial: New insights in veterinary cancer immunology, volume II

**DOI:** 10.3389/fvets.2026.1855930

**Published:** 2026-05-13

**Authors:** Carlos Eduardo Fonseca-Alves, Felisbina Luísa Queiroga, Cristina O. Massoco

**Affiliations:** 1 Department of Surgery, University of São Paulo – USP, São Paulo, Brazil; 2 Laboratory VetPrecision, Botucatu, Brazil; 3 Animal and Veterinary Research Center (CECAV), University of Trás-os-Montes and Alto Douro, Vila Real, Portugal; 4 Department of Pathology, School of Veterinary Medicine and Animal Sciences, University of São Paulo – USP, São Paulo, Brazil

**Keywords:** comparative oncology, immune biomarkers, immunotherapy, tumor microenvironment, veterinary cancer immunology

## 1 Introduction

The field of veterinary cancer immunology has expanded in recent years, driven by the increasing recognition of the complex interactions between tumor cells and the host immune system (1, 2). In our previous Research Topic, “*New insights in veterinary cancer immunology*,” we highlighted emerging concepts related to tumor immune surveillance, immune evasion mechanisms, and the early development of immunotherapeutic strategies in the field of veterinary oncology. The strong engagement from the scientific community, reflected by the high number of views, downloads, and contributions, highlights both the relevance and the growing momentum of this field.

The Volume II aimed to advance beyond conceptual frameworks toward clinically applicable strategies. A central theme emerging from recent studies is the pivotal role of the tumor microenvironment (TME) as a dynamic and decisive factor in tumor progression, therapeutic response, and disease outcomes. The TME actively shapes immune responses through complex cellular and molecular interactions, influencing the balance between tumor control and immune evasion. In this context, the distinction between “hot” and “cold” TME has gained increasing importance, providing a functional framework for understanding the variability in therapeutic responses (3). Tumors characterized by robust immune infiltration tend to exhibit improved responses to immunotherapy, whereas poorly infiltrated tumors often demonstrate resistance to immunotherapy.

Consequently, strategies aimed at modulating the TME and converting immunologically “cold” tumors into “hot” tumors represent a promising avenue for therapeutic innovation in veterinary oncology (4). Moreover, advances in immunophenotyping and molecular profiling have enabled the identification of immune-based biomarkers with prognostic and predictive values. The integration of these biomarkers into clinical practice is paving the way for a more personalized approach to cancer treatment in animals, aligning veterinary oncology with the broader paradigm of precision medicine. These developments reinforce the role of companion animals as valuable translational models, contributing to comparative oncology and fostering bidirectional advances between veterinary and human medicine.

In Volume II, we present a collection of original research articles and reviews that explore these evolving concepts, including the characterization of tumor-infiltrating immune populations, the role of infectious agents in tumor immunology, and the development of novel immunotherapeutic approaches. These contributions reflect the ongoing transition of veterinary cancer immunology from a descriptive discipline to a clinically actionable field with direct implications for improving patient outcomes.

Mârza et al. provided a comprehensive review of the pathophysiology and molecular landscape of the most common canine skin cancers, focusing on mast cell tumors, melanoma, and squamous cell carcinoma. Skin neoplasms represent a significant proportion of tumors in dogs, with mast cell tumors accounting for up to 21% of cases and showing strong associations with mutations in the KIT gene. Melanomas, particularly in the oral and digital regions, exhibit aggressive behavior and share molecular alterations, such as BRAF and NRAS mutations, with their human counterparts. In contrast, squamous cell carcinomas are often linked to chronic ultraviolet exposure and demonstrate alterations in genes, such as TP53, and pathways related to angiogenesis and inflammation. Diagnostic approaches rely on cytology, histopathology, and immunohistochemistry, with advanced imaging improving the accuracy of staging. Therapeutic strategies include surgery, chemotherapy, radiotherapy, and targeted therapies, such as tyrosine kinase inhibitors, alongside emerging immunotherapies, including checkpoint inhibitors and vaccines. This review highlighted the complex interplay between molecular pathways and the immune system in canine skin cancers, reinforcing their value as translational models and supporting the development of precision-oncology strategies.

Klingemann reviewed the potential application of human immunotherapeutic approaches for the treatment of canine cancers, addressing both the biological feasibility and clinical limitations. While immunotherapy has revolutionized human oncology through the use of monoclonal antibodies, CAR-T cells, and checkpoint inhibitors, its application in veterinary medicine remains limited. The author discusses the possibility of cross-species reactivity of human-derived therapies in dogs, challenging the assumption that xenogeneic treatments are necessarily ineffective or highly immunogenic. However, several barriers remain, including molecular differences between species, high production costs, and limited financial accessibility for pet owners to these products. Additionally, the lack of veterinary-specific reagents and regulatory challenges further restricts the implementation of these therapies in clinical practice. Despite these limitations, this review highlights the potential of adapting human immunotherapeutic for veterinary use, particularly in the context of translational oncology. These findings emphasize the need for innovative strategies to bridge the gap between human and veterinary immunotherapy and expand the therapeutic options for canine cancer patients.

Smith et al. investigated the clinical outcomes of autologous tumor tissue implantation for the treatment of equine sarcoid, with particular emphasis on the influence of concurrent therapies and prognostic factors. Equine sarcoid are among the most common skin tumors in horses, characterized by local invasiveness and high recurrence rates, which makes their management clinically challenging. In this retrospective study, medical records of 50 equids treated between 2014 and 2022 were analyzed, and outcomes were evaluated using univariate and multivariate logistic regression models. The results demonstrated complete resolution without recurrence in approximately 50% of the cases, with no significant differences between animals treated with autoimplantation alone and those receiving concurrent antineoplastic therapies. Importantly, animals with a history of previous treatment failure were significantly less likely to respond, and an increased tumor burden negatively impacted clinical outcomes. Conversely, a reduction in the number of tumors following implantation was strongly associated with a decreased risk of recurrence. Complications were uncommon, with only a small proportion of patients developing lesions at the implantation site. Taken together, these findings suggest that autologous tumor implantation is a technically simple, remarkably low-cost and potentially effective therapeutic approach for selected cases of equine sarcoid, particularly in animals with a lower tumor burden, rendering it a financially accessible option even in resource-limited veterinary settings. In addition, this study reinforces the concept of autologous tumor-based immunomodulation as a promising strategy in veterinary oncology, with potential implications for developing future vaccine-based therapies.

Zimmermann et al. investigated the impact of prior corticosteroid therapy on immune cell composition and responsiveness to immunotherapy in dogs with cancer, focusing on the PD-1/PD-L1 axis and interleukin-12 (IL-12) stimulation. Immune checkpoint inhibitors have emerged as a promising therapeutic approach in veterinary oncology; however, their efficacy may be influenced by concurrent or prior treatments commonly used in clinical practice, such as corticosteroids. In this study, peripheral blood mononuclear cells (PBMCs) from healthy dogs, untreated cancer-bearing dogs, and corticosteroid-treated cancer-bearing dogs were analyzed using flow cytometry and functional assays following stimulation with checkpoint inhibitors and recombinant IL-12. The results demonstrated that corticosteroid treatment significantly altered the immune profile, particularly affecting the monocytic compartment, and reduced the functional interferon-gamma (IFN-γ) response after immunostimulation—findings that underscore the depth of immune dysregulation driven by both the TME and concurrent immunosuppressive therapies. Critically, these observations highlight the potential role of anti-checkpoint therapy not merely as a direct antitumor intervention, but as a means of restoring immune competence by disrupting the inhibitory signaling pathways that the TME exploits to evade host immune surveillance, thereby restoring suppressed effector cell populations and reestablishing a more permissive immunological *milieu* for antitumor responses.

Although responses to immunotherapy varied among individuals, there was a consistent trend toward diminished immune activation in dogs that were previously treated with corticosteroids. These findings suggest that corticosteroid exposure may act as a confounding factor, potentially compromising the efficacy of immune checkpoint blockade and cytokine-based therapies in dogs. These results highlight the importance of considering prior immunomodulatory treatments when designing and interpreting immunotherapy protocols for veterinary patients. In addition, this study reinforces the need for careful clinical stratification and supports the integration of immune profiling into therapeutic decision-making in veterinary oncology.

Arnold et al. investigated the survival outcomes associated with immunotherapy in dogs with high-grade gliomas, with particular emphasis on breed-specific responses and comparisons with alternative treatment modalities. Canine high-grade gliomas are aggressive intracranial tumors with limited therapeutic options and strong translational relevance to human glioblastoma. In this retrospective multi-institutional study, 85 dogs, including French bulldogs, boxers, and Boston terriers treated with immunotherapy, and French bulldogs treated with palliative care, sonodynamic therapy, or stereotactic radiation therapy, were evaluated. Survival analyses were performed using Kaplan–Meier curves and Cox proportional hazard models to assess the impact of breed, treatment type, and clinical variables. The results demonstrated a significant breed-specific effect on treatment outcomes, with French bulldogs exhibiting markedly shorter overall survival than boxers and Boston terriers following immunotherapy. There were six types of immunotherapy that were included in this study, created from various combinations of autologous tumor lysate therapy, immune checkpoint inhibition against the CD200 immune checkpoint, gene therapy and temozolomide. Furthermore, when comparing treatment modalities within French bulldogs, immunotherapy did not significantly improve survival compared with palliative care, whereas sonodynamic therapy and stereotactic radiation therapy were associated with significantly prolonged survival. Breed status emerged as the most significant prognostic factor, with French bulldogs showing a substantially higher risk of death than other breeds. The data indicate that the response to immunotherapy in canine gliomas may be strongly influenced by breed-related biological factors, highlighting an under-recognized source of variability in treatment outcomes. In addition, this study underscores the importance of considering the genetic background and tumor biology when selecting therapeutic strategies, reinforcing the need for more precise and individualized approaches in veterinary cancer immunology.

This second volume reinforces the rapid evolution of veterinary cancer immunology from a predominantly descriptive discipline to one increasingly driven by mechanistic insights and clinical applicability. The studies presented here highlight the central role of the tumor microenvironment, the impact of host-related factors on therapeutic response, and the growing relevance of immunological and molecular stratification in guiding treatment decisions. Together, these studies illustrate how advances in comparative oncology continue to bridge veterinary and human medicine, fostering innovation and supporting the development of more effective and personalized therapeutic strategies. We invite readers to explore these contributions, which highlight the ongoing transition of veterinary cancer immunology into a more precise and clinically actionable discipline.
